# Transcutaneous Nerve Electrostimulation (TENS) in Pain Relief During Labor: A Scope Review

**DOI:** 10.1055/s-0042-1742290

**Published:** 2022-02-25

**Authors:** Carla Cristina Silveira dos Reis, Leandro da Cunha Dias, Lorena Bezerra Carvalho, Lourivaldo Bispo Alves Junior, Aline Mizusaki Imoto

**Affiliations:** 1 Fundação de Ensino e Pesquisa em Ciências da Saúde (FEPECS), Escola Superior de Ciências da Saúde, Brasília, DF, Brazil

**Keywords:** transcutaneous nervous electrical stimulation, labor pain, birth work, estimulação eléctrica nervosa transcutânea, dores de parto, trabalho de parto

## Abstract

**Objective** To map health evidence on the effectiveness of transcutaneous nerve electrostimulation (TENS) therapy in pain relief during delivery.

**Methods** This is a scoping review in the PubMed, LILACS, Cochrane, VHL, PEDRO, and SciELO databases, through the descriptors *electric stimulation, transcutaneous* and *labor, obstetric* and their synonyms.

**Results** A total of 263 studies were identified, of which 54 duplicates were excluded. After sorting by titles and abstracts, there were 24 articles for reading, remaining 6. The six studies evaluated the reduction of pain through the visual analogue scale (VAS).

**Conclusion** The findings indicate that the use of TENS as a nonpharmacological strategy for pain relief in labor has positive results.

## Resumo

**Objetivo** Mapear evidências em saúde sobre a eficácia da terapia por estimulação elétrica nervosa transcutânea (TENS, na sigla em inglês) no alívio da dor durante o parto.

**Métodos** Trata-se de uma revisão de escopo nas bases de dados PubMed, LILACS, Cochrane, VHL, PEDRO e SciELO, através dos descritores *estimulação eléctrica, transcutânea* e *trabalho, obstetrícia* e os seus sinônimos.

**Resultados** Foram identificados 263 estudos, dos quais 54 duplicados foram excluídos. Após a classificação por títulos e resumos, 24 artigos foram selecionados para leitura, restando 6. Os seis estudos avaliaram a redução da dor através da escala visual analógica (EVA).

**Conclusão** Os resultados indicam que a utilização de TENS como estratégia não farmacológica para o alívio da dor no parto tem resultados positivos.

## Introduction

Pain during labor is one of the most intense types of pain. This pain is a complex, subjective, and multidimensional phenomenon, which can vary from one person to another, being a physiological response to the sensory stimuli generated mainly by uterine contraction. 1 2 3 Women who experience high levels of pain during childbirth are at an increased risk of complications. 2

In order to minimize the discomfort of pain during childbirth, humanized obstetric care should be provided with access by the parturient to pharmacological and nonpharmacological resources for pain relief in this process. 3

Alternative nonpharmacological methods are recommended by the World Health Organization (WHO) for the care of normal delivery, classifying them as "behaviors that are clearly useful and that should be encouraged”. 4

Although there is a wide range of alternative options, there is still a preference among medical professionals to use in labor anesthetic drugs for pain relief that can cause undesirable effects, such as halting the progression of childbirth and fetal depression. This attitude seems to be contrary to the current expectations of society, which demand safe techniques that guarantee the reduction of labor pain, allowing them to participate actively throughout the work, without any unfavorable repercussions for the mother and the newborn. 4 5

In this perspective, complementary therapy practices have been used both in the public and in the private health network in order to provide assistance in labor, being a strategy for reducing pain, stress, and cesarean rates, reflecting in the quality of obstetric care provided. 6

Alternative methods of pain control provide women with the opportunity to have a positive view of the special moment that is the arrival of the child, increasing satisfaction with their experience in labor. Among the nonpharmacological methods of pain relief in childbirth is the application of transcutaneous nerve electrostimulation (TENS). 2 3

Transcutaneous nerve electrostimulation is a noninvasive, safe physical therapy resource offered to parturients by the physiotherapist during labor. 7 8 The technique basically consists of administering low-voltage electrical impulses or stimuli through electrodes placed on the skin to reduce the painful sensation of labor, delaying or avoiding the need to use pharmacological methods. 6

A preliminary survey, carried out in 2009 in the Cochrane Database of Systematic Reviews, revealed that TENS does not seem to have any impact (positive or negative) in reducing pain caused by labor due to the evidence being often obtained with weak and inconsistent methods. 9

Considering the importance of nonpharmacological methods for a successful experience in labor and the need to evaluate the effectiveness of alternative methods, the present work aims to carry out a systematic search strategy (scoping review) in the main databases, over the past 10 years, to map new health evidence regarding the effectiveness of TENS therapy in pain relief during labor.

## Methods

A scoping review is a secondary study with the objective of mapping the literature, clarifying the main concepts on the question formulated and presenting the types of evidence that may support the practice on the subject. 10 11

This review addressed the following research question: what is the available scientific evidence on the effectiveness of transcutaneous nerve electrical stimulation (TENS) as a non-pharmacological therapy for pain relief during labor? The question and the main elements of the search for this study were elaborated from the PCC (Population, Concept and Context) strategy 11 . In this research, the following definitions were used: P - pregnant women, C - TENS and C - labor.

The search was performed electronically in the PubMed, Latin American and Caribbean Center Health Sciences Literature (LILACS ), Cochrane, Virtual Health Library (VHL), PEDRO, and SciELO databases by means of the descriptors and their synonyms, according to the Health Sciences Descriptors (DeCS/Mesh) with the combination by means of the Boolean terms AND and OR. The search was performed in July 2021 and the descriptors *electric stimulation, transcutaneous* and *labor, obstetric* were used, as well as their synonyms.

Clinical trials that addressed the TENS intervention with pain relief during labor as an outcome published in the last 10 years (covering the period from 2011 to 2021) in Portuguese, English, and Spanish were included,

The exclusion criteria were articles that did not focus on the use of TENS during labor to relieve pain, that examined the use of TENS for pain relief after cesarean section, that analyzed the effects of TENS on the strength of uterine contractions during induction of labor, that were performed for methodological reasons, that were not clinical trials, and those which we did not obtain access to the full text.

## Results

**Fig. 1:**
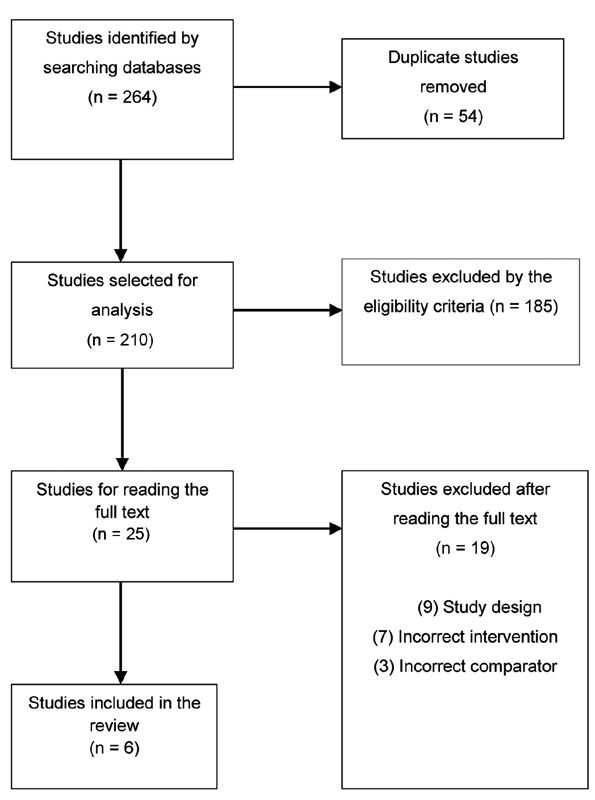
Flowchart of electronic search and inclusion of articles.

A total of 264 studies were identified. Of this total, 54 duplicate articles were excluded, resulting in 209 articles for analysis. After sorting by titles and abstracts according to the eligibility criteria, 24 articles were left to be read in full text, with 19 articles excluded at this stage. Finally, six articles were selected and included for answering the guiding questions of the present scoping review ( Figure 1 ). The entire selection process of the articles was carried out through the site https://www.covidence.org .

Among the studies included, 2 (33,33%) were published in 2010, 1 (16,66%) in 2016, 1 (16,66%) in 2017, 1 (16,66%) in 2018, and 1 (16,66%), the most current to be included, was published in 2021.

The 2 studies published in 2010 carried out nonrandomized controlled clinical trials evaluating the effect of TENS on pain relief in labor, dividing the participants into 2 groups, 1 group receiving TENS as an intervention and the other being a control group. 12 13 The first study evaluated 20 parturients and found that 80% of pregnant women who used TENS felt strong pain relief during labor. 12 The second investigated 305 women and found that there was 68% of pain relief during labor in the group treated with TENS. 13 Both studies evaluated pain reduction using the visual analogue scale (VAS).

In 2016, a randomized clinical trial, blinded and with blind allocation, with 46 parturients was carried out. It was divided into an experimental group, with the application of TENS, and a control group, in which physical therapists guided and answered questions from parturients, in addition to routine obstetric care. 14 After the interventions in both groups, another investigator evaluated the pain through the VAS. Transcutaneous nerve electrostimulation reduced pain in the experimental group, decreasing an average of 11 mm (standard deviation [SD] = 18) until the end of the intervention. Through the VAS, 69% of the control group and 70% of the experimental group classified pain as 37 before the intervention. After the intervention, 34% of the experimental group classified pain as 37, as opposed to 83% of the control group, showing a significant reduction in pain during labor (RR: 0.42; 95% confidence interval [CI]: 0.23–0.76).

In the 2017 randomized, nonblind clinical trial, 90 nulliparous women were included and divided into the following groups: intervention (TENS activated), placebo (TENS deactivated), and control (TENS not used), each group consisting of 30 participants. One hour after the intervention, pain intensity was significantly lower in the experimental group compared with the placebo and control groups. The mean pain intensities assessed by the VAS 1 hour after the intervention were 6.4 (SD = 2.14), 8.4 (SD = 1.38), and 8.2 (SD = 1.6), for the experimental, placebo, and control groups, respectively. 15

The 2018 study conducted a randomized, double-blind, placebo-controlled clinical trial looking at the effect of different doses of TENS for pain relief during labor. 2 In this study, 63 women were recruited, divided into active TENS 1 ( *n* = 21), active TENS 2 ( *n* = 21), and placebo ( *n* = 21) groups. The active TENS 2 group obtained the best performance in pain reduction, with clinically relevant results in the VAS (- 2.9; 95%CI: - 4.1– - 1.6; *p* < 0.001). There was more satisfaction in the active TENS groups than in the placebo group. The analysis between the groups highlighted a significant decrease in pain, measured on the VAS, in the active TENS 2 group compared with the TENS 1 group and also compared with the placebo group.

The most recent study, from 2021, 16 conducted a single-blinded randomized clinical trial with 326 low-risk pregnant women who anticipated spontaneous vaginal delivery. The experimental group had 161 participants and the control group had 165 participants. The VAS was used for pain assessment, and high-tech TENS was used at 30, 60, and 120 minutes and from 2 to 24 hours after delivery. The experimental group had statistically significantly lower mean VAS scores at different times and the experimental group showed statistically significant shorter duration of the active phase of labor than the control group. The authors concluded that the use of TENS had satisfactory results both for reducing pain and for shortening the active phase of labor.

Tables 1 and 2 present the articles according to authors, year of publication, main objectives, intervention, main results, and conclusions.

**Table 1:** Description of the articles included in the scoping review

Authors and year	Objective	Intervention	Results	Conclusions
Abreu et al. (2010) 12	To evaluate the effectiveness of TENS in pain relief during labor.	Experimental group: Application of TENS by means of a pair of electrodes at the level of the T10-L1 vertebrae and another pair at the level of S2-S4. Control group: TENS with subliminal stimulus.	TENS provided pain relief in 80% of the cases, assessed by the VAS. There were no cases of pain relief in the control group.	TENS is effective in relieving pain during labor.
Peng et al. (2010) 13	To investigate the effectiveness of TENS to reduce pain in labor when applied to four specific acupuncture points.	Experimental group: Application of TENS in four specific acupuncture points (LI4, PC6, BL19, and BL21). Control group: without any intervention.	There was 68% of pain relief during labor in the group treated with TENS, measured by the VAS	The application of TENS to acupuncture points LI4, PC6, BL19, and BL21 is an effective, noninvasive method for pain control during labor.
Santana et al. (2016) 14	To assess whether TENS relieves pain or changes its location in the first phase of labor and whether TENS delays the request for neuraxial analgesia.	Experimental group: Application of TENS by means of a pair of electrodes at the level of the T10-L1 vertebrae and another pair at the level of S2-S4. Control group: physiotherapist for guidance and answer questions.	TENS reduced pain in the experimental group, assessed by the VAS, but did not change its location or distribution. Pharmacological analgesia was postponed for 5 hours after TENS intervention, compared with the control group.	TENS significantly reduces pain in labor and postpones the need for pharmacological analgesia.
Shahoei et al. (2017) 15	To investigate the effect of TENS on labor pain among nulliparous women.	Experimental group: Application of TENS by means of a pair of electrodes at the level of the T10-L1 vertebrae and another pair at the level of S2-S4. Placebo group: TENS off (no electrical stimulus). Control group: no TENS, just routine care and guidance.	TENS provided a significant reduction in pain during labor and also 4 hours later, assessed by the VAS. TENS had no placebo effect.	TENS reduces pain during labor and 4 hours afterwards.
Báez-Suárez et al. (2018) 2	To investigate the effect of pain relief by applying TENS in labor.	Experimental group: Application of TENS by means of a pair of electrodes at the level of the T10-L1 vertebrae and another pair at the level of S2-S4. Placebo group: TENS off (no electrical stimulus).	TENS provided a greater degree of pain relief in the active TENS group than in the placebo TENS group.	TENS is a safe and effective nonpharmacological option for pain relief during labor.
Njogu et al. (2021) 16	To determine the effects of TENS therapy in the first stage of labor.	Experimental group: Application of TENS by means of a pair of electrodes at the level of the T10-L1 vertebrae and S2-S4, in addition to a pair in each arm. Control group: routine obstetric care.	The experimental group had lower mean pain scale scores than the control group and shorter duration of the active phase of labor.	TENS can be used as a nonpharmacological therapy to reduce pain and shorten the active phase of labor.

Abbreviation: TENS, transcutaneous nerve electrical stimulation; VAS, visual analogue scale.

**Table 2:** Characteristics of the studies included in the present review

Authors	Participants	Age range	Study methodology	Blinding	Subject allocation	Eligibility criteria
Abreu et al. (2010) 12	20	18–26	Nonrandomized controlled trial	No	Numeration of medical records (medical records with even endings were allocated to the TENS group, and those with odd endings to the control group)	Parturients between 18 and 30 years old; term pregnancy; active stage of labor; single viable fetus in cephalic presentation.
Peng et al. (2010) 13	305	24–30	Nonrandomized controlled trial	No	Pregnant women who requested analgesia were enrolled in the TENS group and others were assigned to the control group	Pregnant women who dismissed epidural analgesia; term pregnancy; primiparous with singleton; fetal cephalic presentation; active stage of labor; have no obstetric complications.
Santana et al. (2016) 14	46	18–21	Randomized controlled trial	Evaluator	Computer-generated random assignment list	Primiparous women do not have obstetric complications; term pregnancy; single fetus in cephalic position; spontaneous onset of labor; no use of oxytocin or other medications from hospital admission until randomization.
Shahoei et al. (2017) 15	90	16–36	Randomized controlled trial	No		Primiparous; single fetus in cephalic position; pregnancy age of 38-42 weeks; active phase of labor; intact membranes.
Báez-Suárez et al. (2018) 2	63	22–35	Randomized controlled trial	Double-blind	Computerized random number generator	Age > 18 years old; women with a low-risk pregnancy; 37–42 weeks gestation age; single fetus.
Njogu et al. (2021) 16	326	18–29	Randomized controlled trial	Evaluator	Computer-generated list	37–42 weeks gestation age; primipara and multipara with no complications; active stage of labor; single viable fetus in cephalic presentation.

Abbreviation: TENS, transcutaneous nerve electrical stimulation.

## Discussion

In the present scoping review, the results of the six studies were favorable regarding the use of TENS in pain relief during labor. The articles included in the present review are described below, outlining the main outcome, which is pain relief.

Most studies (75%) included in an integrative review conducted by Mafetoni et al. 17 in which they applied TENS for pain relief during labor demonstrated increased pain reduction responses compared with the placebo group, corroborating the findings of the present study.

In the open and randomized clinical trial conducted by Orange et al. 5 that evaluated 22 women in labor, it was found that the application of TENS in the early stages of labor delays the need for additional analgesic techniques, confirming the findings of the present scoping review.

The main advantage of using TENS in labor would be the absence of the need to use drugs or the shorter time of exposure of the mother and fetus to drugs used for pain relief, which could reduce the incidence of undesirable effects, such as halting the progression of childbirth and fetal depression. 5

Knobel et al. 18 conducted a controlled, randomized and triple-blind (neither the parturients, the health team, nor the researcher knew which group each participant belonged to) with 60 parturients, using TENS to relieve pain during labor. They concluded that there was significant pain relief in more than half of the women who received real stimulation than in those who received placebo stimulation. 18

Angelo et al. 19 carried out a systematic review on the effects of physical therapy resources applied to patients suffering from pain during labor. They found a variety of methods, such as massage therapy, TENS, ball exercises, immersion bath, breathing exercises, acupuncture, ambulation, mobility, and shower. They concluded that, in their majority, nonpharmacological methods contributed in a beneficial way to reducing the pain of the parturient. 19

However, the systematic review by Dowswell et al., 9 in which 17 studies were analyzed involving 1,466 women, concluded that there is no strong evidence that women who received TENS had reduced pain in childbirth compared with control groups. These findings differ from the results of the present scoping review. 9

In line with the findings of Dowswell et al., 9 the systematic review by Mello et al. 1 confirms that there was no statistically significant difference between groups in pain relief during labor (combined RR = 1.09; 95%CI = 0.72–1.65), which indicates limitations in the evidence that TENS reduces pain during labor. 1 9

It is observed that, over the years, clinical trials developed to evaluate the effectiveness of TENS in reducing pain during labor have limitations in terms of methodological rigor. The controlled clinical trial of Abreu et al. 12 identified by the present review, used the criteria of numbering medical records for the allocation of participants, presenting a high risk of selection bias and of being able to interfere in the results. It is known that it is extremely important to ensure adequate randomization and confidentiality in the allocation of patients, which is the best way to minimize selection bias and to ensure more robust evidence.

The clinical trial by Njogu et al. 16 used a simple random assignment technique in a 1:1 ratio with researcher blinded to one group; the workers and researchers in the gynecology and obstetrics department knew the treatment group, and only the statisticians analyzed the blinded data. However, the study has limitations that may compromise the reliability of the results, such as simple blinding and inclusion of primiparous and multiparous women without specifying the proportionality of distribution in the groups. 16

There is also weakness in relation to blinding clinical trials, where only the studies of Santana et al., 14 Báez-Suárez et al., 2 and Njogu et al. 16 performed some kind of blinding, which compromises the power of evidence in these studies. 2 14 16 17 18 19

Despite the mentioned limitations, it is worth highlighting the importance of the studies carried out to evaluate the effectiveness of nonpharmacological methods that are encouraged by the WHO because they are strategies used in labor to increase tolerance to pain and to reduce undesirable effects related to drug therapy, and should be further explored with a deeper analysis of the context in which they were developed.

## Conclusion

After an updated, transparent, and thorough search, and the analysis of the studies with the best levels of evidence, the findings of the present scoping review indicate that the use of TENS as a nonpharmacological strategy for pain relief in labor has positive results compared with the placebo intervention group, and it is possible to observe its effects even hours after its use; however, most studies still present inadequate methodological rigor, presenting a risk of bias regarding the evaluation of the effectiveness of the therapy. Another point is the method used for pain assessment in the studies, because pain is a personal experience, which makes it difficult to define and measure it. The perception of pain is individual, subjective, and influenced by the intensity and duration of exposure, the physical and emotional condition of the woman, her previous experiences, cultural and environmental factors, and several aspects can change the assessment of pain and influence the results of the studies. Therefore, for a better evaluation of the effects of this therapy, it is suggested the design of triple-blinded randomized clinical trials with confidentiality of allocation, showing clarity in the parameters used, with an appropriate selection of participants in order to reduce the differences related to experiences with pain in labor, and associated with other scales for pain assessment in order to measure more reliably the relief of pain in parturients. The present scoping review allowed mapping the scientific evidence available in the last 10 years that describe the action of TENS as a nonpharmacological therapy for pain relief during labor. New, more elaborate studies regarding the method and that may compare TENS with other nonpharmacological pain control therapies that have proven results are suggested.
